# *catena*-Poly[[μ-bromido-(μ-hydroxydi-2-pyridylmethano­lato-κ^4^ *N*,*O*:*O*,*N*′)dicopper(II)(*Cu*—*Cu*)]-di-μ-bromido]

**DOI:** 10.1107/S1600536808024197

**Published:** 2008-08-06

**Authors:** Matthias Zeller, Barry L. Westcott, Kristin M. Kopp-Vaughn, Allen D. Hunter

**Affiliations:** a Department of Chemistry, Youngstown State University, Youngstown, OH 44555, USA; b Department of Chemistry and Biochemistry, Central Connecticut State University, New Britain, CT 06050, USA

## Abstract

The title complex, [Cu_2_Br_3_(C_11_H_9_N_2_O_2_)]_*n*_, was one of three isolated by slow evaporation of an acetonitrile reaction mixture of CuBr_2_ with di-2-pyridyl ketone (1:1 molar ratio). The title complex contains a 2:1 metal-to-ligand ratio of copper(II) with the hydrated form of the ligand, di-2-pyridylmethane­diol. The two copper centers are bridged by a bromide ion and the alk­oxy O atom, and the Cu—Cu distance is 2.9801 (5) Å. The dimeric units are further linked by bromide ions, leading to a two-dimensional extended bridged structure. O—H⋯O hydrogen bonds are present in the crystal structure.

## Related literature

Apart from the title complex, two others were isolated from the reaction mixture and structurally characterized. One was identical to that of Parker *et al.* (2000 ▶), the other is reported in the following paper by Westcott *et al.* (2008 ▶). For other related structures, see: Wang *et al.* (1986 ▶); Mariezcurrena *et al.* (1999 ▶).
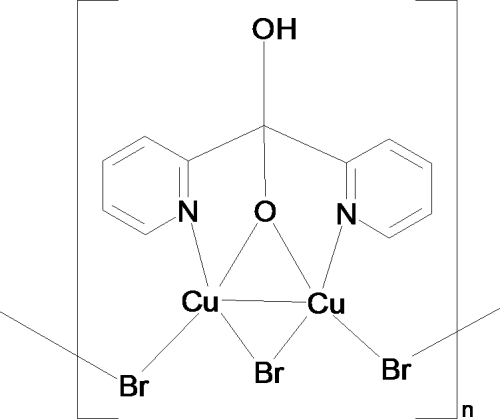


## Experimental

### 

#### Crystal data

[Cu_2_Br_3_(C_11_H_9_N_2_O_2_)]
*M* *_r_* = 568.01
Triclinic, 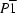

*a* = 8.7708 (7) Å
*b* = 9.6018 (8) Å
*c* = 10.1839 (8) Å
α = 73.7060 (10)°
β = 70.8520 (10)°
γ = 63.9280 (10)°
*V* = 718.28 (10) Å^3^
*Z* = 2
Mo *K*α radiation
μ = 11.30 mm^−1^
*T* = 100 (2) K
0.39 × 0.19 × 0.08 mm

#### Data collection

Bruker SMART APEX CCD diffractometer
Absorption correction: multi-scan (*SADABS* in *SAINT-Plus*; Bruker, 2003 ▶) *T*_min_ = 0.122, *T*_max_ = 0.424
7397 measured reflections
3537 independent reflections
3305 reflections with *I* > 2σ(*I*)
*R*_int_ = 0.021

#### Refinement

*R*[*F* ^2^ > 2σ(*F* ^2^)] = 0.023
*wR*(*F* ^2^) = 0.061
*S* = 1.09
3537 reflections
182 parameters
H-atom parameters constrained
Δρ_max_ = 1.02 e Å^−3^
Δρ_min_ = −0.60 e Å^−3^

### 

Data collection: *SMART* (Bruker, 2002 ▶); cell refinement: *SAINT-Plus* (Bruker, 2003 ▶); data reduction: *SAINT-Plus*; program(s) used to solve structure: *SHELXTL* (Sheldrick, 2008 ▶); program(s) used to refine structure: *SHELXTL*; molecular graphics: *SHELXTL* and *ORTEP-3* (Farrugia, 1997 ▶); software used to prepare material for publication: *SHELXTL*.

## Supplementary Material

Crystal structure: contains datablocks I, global. DOI: 10.1107/S1600536808024197/fj2131sup1.cif

Structure factors: contains datablocks I. DOI: 10.1107/S1600536808024197/fj2131Isup2.hkl

Additional supplementary materials:  crystallographic information; 3D view; checkCIF report

## Figures and Tables

**Table 1:** Hydrogen-bond geometry (Å, °)

*D*—H⋯*A*	*D*—H	H⋯*A*	*D*⋯*A*	*D*—H⋯*A*
O1—H1⋯O2^i^	0.84	2.33	3.014 (2)	139

Symmetry code: (i) 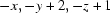.

**Fig. 1.**
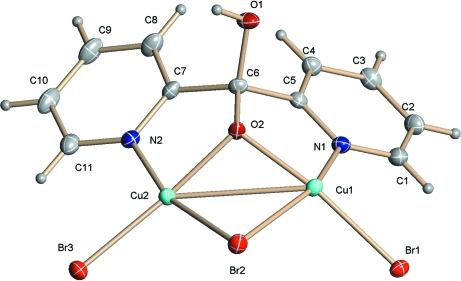
ORTEP-3 (Farrugia, 1997) representation of the asymmetric unit. Ellipsoids are drawn at the 30% probability level.

**Fig. 2.**
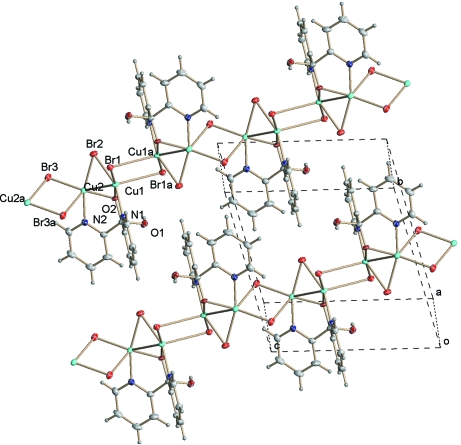
Packing diagram showing the extended structure.

## supplementary crystallographic information

### Comment

The structure of the title compound, is shown below. The complex was one of three Cu-dpkoh complexes isolated from the 1:1 molar mixture of copper(II)bromide and di-2-pyridyl ketone. The title complex was the second isolated from solution. One other complex had been reported previously by Parker, *et al.*, another unique complex is described elsewhere: Westcott, *et al.*(2008).

The complex contains two copper centers that are 2.9801 (5) Å apart and are bridged by the hydrated form of the ligand di-2-pyridylketone. Each copper center is also coordinated to the ligand through one unique nitrogen atom and a by a µ-hydroxyl bridge. Additionally, each Cu center coordinates one bromide ion. The bromide ion then acts as a bridging ligand to the next di-copper unit, leading to a polymeric structure as shown in Figure 2.

### Experimental

Di-2-pyridyl ketone (dpk) was purchased from Aldrich and used as received. Copper(II) bromide hexahydrate was dried in an oven at 110 C for 48 h before use. DPK (1 mmol) and copper(II) bromide (1 mmol) were combined in 40 ml of acetonitirle and stirred for 30 minutes. The resulting green crystals were isolated after 4 days by slow evaporation of the solution.

### Refinement

For structure solution, direct methods were used to locate the initial structural model that consisted of all non-hydrogen atoms. All ligand-based H atoms were added during the refinement stage at idealized positions (C—H 0.95 Å; O—H 0.84 Å). All H atoms were refined with isotropic displacement parameter set equal to 1.5 times the isotropic equivalent value for the attached atom. All non-hydrogen atoms were refined anisotropically.

### Crystal data

**Table:**

[Cu_2_Br_3_(C_11_H_9_N_2_O_2_)]	*Z* = 2
*M**_r_* = 568.01	*F*_000_ = 536
Triclinic, *P*1	*D*_x_ = 2.626 Mg m^−^^3^
Hall symbol: -P 1	Mo *K*α radiation λ = 0.71073 Å
*a* = 8.7708 (7) Å	Cell parameters from 6753 reflections
*b* = 9.6018 (8) Å	θ = 2.4–30.6º
*c* = 10.1839 (8) Å	µ = 11.30 mm^−^^1^
α = 73.7060 (10)º	*T* = 100 (2) K
β = 70.8520 (10)º	Hexagon, green
γ = 63.9280 (10)º	0.39 × 0.19 × 0.08 mm
*V* = 718.28 (10) Å^3^	

### Data collection

**Table:**

Bruker SMART APEX CCD diffractometer	3537 independent reflections
Radiation source: fine-focus sealed tube	3305 reflections with *I* > 2σ(*I*)
Monochromator: graphite	*R*_int_ = 0.021
*T* = 100(2) K	θ_max_ = 28.3º
ω scans	θ_min_ = 2.1º
Absorption correction: multi-scan(SADABS in SAINT-Plus; Bruker, 2003)	*h* = −11→11
*T*_min_ = 0.122, *T*_max_ = 0.424	*k* = −12→12
7397 measured reflections	*l* = −13→13

### Refinement

**Table:**

Refinement on *F*^2^	Secondary atom site location: difference Fourier map
Least-squares matrix: full	Hydrogen site location: inferred from neighbouring sites
*R*[*F*^2^ > 2σ(*F*^2^)] = 0.023	H-atom parameters constrained
*wR*(*F*^2^) = 0.061	*w* = 1/[σ^2^(*F*_o_^2^) + (0.0301*P*)^2^ + 0.561*P*] where *P* = (*F*_o_^2^ + 2*F*_c_^2^)/3
*S* = 1.09	(Δ/σ)_max_ = 0.002
3537 reflections	Δρ_max_ = 1.02 e Å^−^^3^
182 parameters	Δρ_min_ = −0.60 e Å^−^^3^
Primary atom site location: structure-invariant direct methods	Extinction correction: none

### Special details

**Table:**

Geometry. All e.s.d.'s (except the e.s.d. in the dihedral angle between two l.s. planes) are estimated using the full covariance matrix. The cell e.s.d.'s are taken into account individually in the estimation of e.s.d.'s in distances, angles and torsion angles; correlations between e.s.d.'s in cell parameters are only used when they are defined by crystal symmetry. An approximate (isotropic) treatment of cell e.s.d.'s is used for estimating e.s.d.'s involving l.s. planes.
Refinement. Refinement of *F*^2^ against ALL reflections. The weighted *R*-factor *wR* and goodness of fit *S* are based on *F*^2^, conventional *R*-factors *R* are based on *F*, with *F* set to zero for negative *F*^2^. The threshold expression of *F*^2^ > σ(*F*^2^) is used only for calculating *R*-factors(gt) *etc*. and is not relevant to the choice of reflections for refinement. *R*-factors based on *F*^2^ are statistically about twice as large as those based on *F*, and *R*- factors based on ALL data will be even larger.

### Fractional atomic coordinates and isotropic or equivalent isotropic displacement parameters (Å^2^)

**Table:**

	*x*	*y*	*z*	*U*_iso_*/*U*_eq_	
Br2	0.12154 (3)	1.22298 (3)	0.70734 (3)	0.01693 (7)	
Br3	−0.23030 (3)	1.19011 (3)	1.00202 (3)	0.01823 (7)	
Br1	0.57965 (3)	0.99477 (3)	0.65263 (3)	0.01507 (7)	
Cu1	0.32811 (4)	0.96542 (3)	0.64120 (3)	0.01335 (8)	
Cu2	−0.03366 (4)	1.04405 (4)	0.82256 (3)	0.01406 (8)	
O2	0.1101 (2)	0.9459 (2)	0.65750 (18)	0.0130 (3)	
O1	0.1005 (2)	0.7855 (2)	0.53023 (19)	0.0181 (4)	
H1	0.0027	0.8513	0.5168	0.027*	
N1	0.4203 (3)	0.7340 (2)	0.6562 (2)	0.0142 (4)	
C6	0.1192 (3)	0.7966 (3)	0.6588 (3)	0.0136 (5)	
N2	−0.1178 (3)	0.8719 (3)	0.8672 (2)	0.0148 (4)	
C7	−0.0283 (3)	0.7649 (3)	0.7798 (3)	0.0146 (5)	
C5	0.3027 (3)	0.6743 (3)	0.6683 (2)	0.0132 (5)	
C4	0.3512 (3)	0.5141 (3)	0.6783 (3)	0.0165 (5)	
H4	0.2670	0.4726	0.6880	0.020*	
C3	0.5248 (3)	0.4151 (3)	0.6739 (3)	0.0181 (5)	
H3	0.5603	0.3051	0.6801	0.022*	
C11	−0.2540 (3)	0.8573 (3)	0.9709 (3)	0.0194 (5)	
H11	−0.3182	0.9345	1.0310	0.023*	
C1	0.5880 (3)	0.6375 (3)	0.6519 (3)	0.0181 (5)	
H1A	0.6702	0.6812	0.6426	0.022*	
C10	−0.3034 (4)	0.7321 (4)	0.9924 (3)	0.0231 (6)	
H10	−0.3998	0.7232	1.0667	0.028*	
C9	−0.2103 (4)	0.6209 (3)	0.9042 (3)	0.0250 (6)	
H9	−0.2400	0.5328	0.9187	0.030*	
C2	0.6449 (3)	0.4767 (3)	0.6606 (3)	0.0185 (5)	
H2	0.7641	0.4106	0.6575	0.022*	
C8	−0.0737 (4)	0.6390 (3)	0.7948 (3)	0.0213 (5)	
H8	−0.0116	0.5661	0.7305	0.026*	

### Atomic displacement parameters (Å^2^)

**Table:**

	*U*^11^	*U*^22^	*U*^33^	*U*^12^	*U*^13^	*U*^23^
Br2	0.01628 (12)	0.01244 (12)	0.02349 (13)	−0.00619 (10)	−0.00358 (10)	−0.00520 (10)
Br3	0.01571 (12)	0.01965 (14)	0.01898 (13)	−0.00469 (10)	−0.00213 (9)	−0.00841 (10)
Br1	0.01576 (12)	0.01633 (13)	0.01651 (12)	−0.00878 (10)	−0.00493 (9)	−0.00191 (9)
Cu1	0.01234 (14)	0.01089 (15)	0.01833 (16)	−0.00526 (12)	−0.00342 (11)	−0.00347 (12)
Cu2	0.01375 (15)	0.01408 (15)	0.01601 (15)	−0.00656 (12)	−0.00140 (11)	−0.00535 (12)
O2	0.0129 (8)	0.0090 (8)	0.0178 (8)	−0.0051 (6)	−0.0021 (6)	−0.0038 (6)
O1	0.0200 (9)	0.0172 (9)	0.0191 (9)	−0.0040 (7)	−0.0094 (7)	−0.0055 (7)
N1	0.0152 (10)	0.0135 (10)	0.0149 (10)	−0.0050 (8)	−0.0044 (8)	−0.0034 (8)
C6	0.0159 (11)	0.0111 (11)	0.0162 (11)	−0.0057 (9)	−0.0054 (9)	−0.0028 (9)
N2	0.0146 (10)	0.0163 (10)	0.0153 (10)	−0.0068 (8)	−0.0055 (8)	−0.0014 (8)
C7	0.0130 (11)	0.0138 (12)	0.0191 (12)	−0.0063 (9)	−0.0062 (9)	−0.0010 (9)
C5	0.0155 (11)	0.0130 (12)	0.0110 (10)	−0.0056 (9)	−0.0027 (9)	−0.0023 (9)
C4	0.0181 (12)	0.0144 (12)	0.0177 (12)	−0.0074 (10)	−0.0028 (9)	−0.0033 (9)
C3	0.0222 (13)	0.0105 (11)	0.0174 (12)	−0.0041 (10)	−0.0036 (10)	−0.0010 (9)
C11	0.0174 (12)	0.0253 (14)	0.0162 (12)	−0.0098 (11)	−0.0051 (10)	−0.0005 (10)
C1	0.0168 (12)	0.0207 (13)	0.0186 (12)	−0.0064 (10)	−0.0045 (9)	−0.0065 (10)
C10	0.0208 (13)	0.0283 (15)	0.0228 (13)	−0.0165 (12)	−0.0082 (10)	0.0074 (11)
C9	0.0230 (14)	0.0217 (14)	0.0346 (16)	−0.0151 (12)	−0.0106 (12)	0.0044 (12)
C2	0.0170 (12)	0.0158 (12)	0.0205 (13)	−0.0021 (10)	−0.0057 (10)	−0.0051 (10)
C8	0.0191 (12)	0.0146 (12)	0.0329 (15)	−0.0085 (10)	−0.0083 (11)	−0.0021 (11)

### Geometric parameters (Å, °)

**Table:**

Br2—Cu1	2.4592 (4)	N2—C11	1.341 (3)
Br2—Cu2	2.4613 (4)	N2—C7	1.346 (3)
Br3—Cu2	2.3507 (4)	C7—C8	1.386 (4)
Br1—Cu1	2.3862 (4)	C5—C4	1.387 (3)
Br1—Cu1^i^	2.7923 (4)	C4—C3	1.389 (4)
Cu1—O2	1.9513 (17)	C4—H4	0.9500
Cu1—N1	1.981 (2)	C3—C2	1.374 (4)
Cu1—Br1^i^	2.7923 (4)	C3—H3	0.9500
Cu1—Cu2	2.9801 (5)	C11—C10	1.389 (4)
Cu2—O2	1.9386 (17)	C11—H11	0.9500
Cu2—N2	1.979 (2)	C1—C2	1.384 (4)
O2—C6	1.396 (3)	C1—H1A	0.9500
O1—C6	1.410 (3)	C10—C9	1.380 (4)
O1—H1	0.8400	C10—H10	0.9500
N1—C1	1.344 (3)	C9—C8	1.381 (4)
N1—C5	1.344 (3)	C9—H9	0.9500
C6—C5	1.535 (3)	C2—H2	0.9500
C6—C7	1.542 (3)	C8—H8	0.9500
			
Cu1—Br2—Cu2	74.551 (13)	O2—C6—C7	108.55 (19)
Cu1—Br1—Cu1^i^	87.045 (12)	O1—C6—C7	109.37 (19)
O2—Cu1—N1	82.07 (8)	C5—C6—C7	113.9 (2)
O2—Cu1—Br1	172.49 (5)	C11—N2—C7	119.6 (2)
N1—Cu1—Br1	99.71 (6)	C11—N2—Cu2	126.15 (19)
O2—Cu1—Br2	81.11 (5)	C7—N2—Cu2	114.21 (17)
N1—Cu1—Br2	155.54 (6)	N2—C7—C8	121.3 (2)
Br1—Cu1—Br2	94.848 (14)	N2—C7—C6	115.8 (2)
O2—Cu1—Br1^i^	94.28 (5)	C8—C7—C6	122.8 (2)
N1—Cu1—Br1^i^	91.84 (6)	N1—C5—C4	121.0 (2)
Br1—Cu1—Br1^i^	92.955 (12)	N1—C5—C6	115.0 (2)
Br2—Cu1—Br1^i^	107.004 (14)	C4—C5—C6	123.9 (2)
O2—Cu1—Cu2	39.84 (5)	C5—C4—C3	119.0 (2)
N1—Cu1—Cu2	103.54 (6)	C5—C4—H4	120.5
Br1—Cu1—Cu2	132.975 (15)	C3—C4—H4	120.5
Br2—Cu1—Cu2	52.757 (10)	C2—C3—C4	119.8 (2)
Br1^i^—Cu1—Cu2	126.084 (13)	C2—C3—H3	120.1
O2—Cu2—N2	82.40 (8)	C4—C3—H3	120.1
O2—Cu2—Br3	172.22 (6)	N2—C11—C10	121.6 (3)
N2—Cu2—Br3	99.71 (6)	N2—C11—H11	119.2
O2—Cu2—Br2	81.30 (5)	C10—C11—H11	119.2
N2—Cu2—Br2	163.70 (6)	N1—C1—C2	122.2 (2)
Br3—Cu2—Br2	96.420 (15)	N1—C1—H1A	118.9
O2—Cu2—Cu1	40.15 (5)	C2—C1—H1A	118.9
N2—Cu2—Cu1	113.10 (6)	C9—C10—C11	119.0 (3)
Br3—Cu2—Cu1	142.336 (15)	C9—C10—H10	120.5
Br2—Cu2—Cu1	52.692 (10)	C11—C10—H10	120.5
C6—O2—Cu2	117.17 (15)	C10—C9—C8	119.3 (3)
C6—O2—Cu1	117.19 (14)	C10—C9—H9	120.3
Cu2—O2—Cu1	100.01 (8)	C8—C9—H9	120.3
C6—O1—H1	109.5	C3—C2—C1	118.3 (2)
C1—N1—C5	119.6 (2)	C3—C2—H2	120.8
C1—N1—Cu1	124.95 (18)	C1—C2—H2	120.8
C5—N1—Cu1	115.39 (17)	C9—C8—C7	119.2 (3)
O2—C6—O1	111.3 (2)	C9—C8—H8	120.4
O2—C6—C5	109.75 (19)	C7—C8—H8	120.4
O1—C6—C5	104.04 (19)		

Symmetry codes: (i) −*x*+1, −*y*+2, −*z*+1.

### Hydrogen-bond geometry (Å, °)

**Table:**

*D*—H···*A*	*D*—H	H···*A*	*D*···*A*	*D*—H···*A*
O1—H1···O2^ii^	0.84	2.33	3.014 (2)	139

Symmetry codes: (ii) −*x*, −*y*+2, −*z*+1.

## References

[bb1] Bruker (2002). *SMART* Bruker AXS Inc., Madison, Wisconsin, USA.

[bb2] Bruker (2003). *SAINT-Plus* Bruker AXS Inc., Madison, Wisconsin, USA.

[bb3] Farrugia, L. J. (1997). *J. Appl. Cryst.* **30**, 565.

[bb4] Mariezcurrena, R. A., Mombrú, A. W., Suescun, L., Kremer, E. & González, R. (1999). *Acta Cryst.* C**55**, 1989–1991.

[bb5] Parker, O. J., Aubol, S. L. & Breneman, G. L. (2000). *Polyhedron*, **19**, 623–626.

[bb6] Sheldrick, G. M. (2008). *Acta Cryst.* A**64**, 112–122. 10.1107/S0108767307043930 18156677

[bb7] Wang, S.-L., Richardson, J. W. Jr, Briggs, S. J., Jacobson, R. A. & Jensen, W. P. (1986). *Inorg. Chim. Acta*, **111**, 67–72.

[bb8] Westcott, B. L., Kopp-Vaughn, K. M., Daniels, L. M. & Zeller, M. (2008). *Acta Cryst.* E**64**, m1122–m1123. 10.1107/S1600536808024203 PMC2960647 21201584

